# Inferring the decision rules that drive co-foraging affiliations in wild mixed-species parrot groups

**DOI:** 10.1098/rstb.2022.0101

**Published:** 2023-06-05

**Authors:** Vanessa Ferdinand, Elle Pattenden, Donald J. Brightsmith, Elizabeth A. Hobson

**Affiliations:** ^1^ Melbourne School of Psychological Sciences, University of Melbourne, Melbourne, VIC 3010, Australia; ^2^ School of Veterinary Medicine and Biomedical Sciences, Texas A&M University, College Station, TX 77843, USA; ^3^ Department of Biological Sciences, University of Cincinnati, Cincinnati, OH 45221, USA

**Keywords:** categorization, coarse-graining, mixed-species groups, social decision-making, species co-presence, social interactions

## Abstract

Animals gathered around a specific location or resource may represent mixed-species aggregations or mixed-species groups. Patterns of individuals choosing to join these groups can provide insight into the information processing underlying these decisions. However, we still have little understanding of how much information these decisions are based upon. We used data on 12 parrot species to test what kind of information each species may use about others to make decisions about which mixed-species aggregations to participate in. We used co-presence and joining patterns with categorization and model fitting methods to test how these species could be making grouping decisions. Species generally used a simpler lower-category method to choose which other individuals to associate with, rather than basing these decisions on species-level information. We also found that the best-fit models for decision-making differed across the 12 species and included different kinds of information. We found that not only does this approach provide a framework to test hypotheses about why individuals join or leave mixed-species aggregations, it also provides insight into what features each parrot could have been using to make their decisions. While not exhaustive, this approach provides a novel examination of the potential features that species could use to make grouping decisions and could provide a link to the perceptive and cognitive abilities of the animals making these minute-by-minute decisions.

This article is part of the theme issue ‘Mixed-species groups and aggregations: shaping ecological and behavioural patterns and processes’.

## 1. Introduction

Many species form mixed-species animal groups through interspecific attraction [1]. These groups bring individuals from multiple species into close contact and can often help individuals increase their foraging efficiency or decrease their risk of predation [1–3]. Understanding why species associate with other species can provide insight into the resource needs of each species, the competitive dynamics operating within a community and the relative costs and benefits of being in close proximity to other species. Some mixed-species groups can be quite stable, either involving the same mix of species or the same individual members of the group [4,5], with individuals remaining together over weeks, months or years. Other mixed-species groups form and break apart on a much shorter timescale over minutes to hours.

Ephemeral associations could provide insight into the types of information individuals use to make decisions about the composition of the mixed-species groups with which they associate. When studied, this has usually been analysed as either interaction rules each species uses to make collective decisions that can be based on simple rules of attraction based on group size (e.g. [4]) or attraction or repulsion between each species (e.g. [1,2]). These patterns are each based on different information: a species using a simple rule of attraction may only need to perceive the number of individuals in a group, while a species that changes its behaviour depending on the presence of individuals of other species needs to recognize each different species, resulting in species-level relationships that predict individual co-presences. However, the species-level characteristics that we often use as researchers may not accurately represent the different ways in which animals themselves categorize each other. Species may use varying types of information about each other to decide whether to join, remain in, or leave a mixed-species aggregation. Individuals may not focus on which particular species is present, but potentially on physical characteristics like size or colour of group members, especially if dilution of predation risk drives these associations.

Potential categorization systems represent different ways that available information can be summarized or *compressed* by individuals. Compression is a form of information reduction that can remove redundancies and noise in observations [6–8]. When compression results in a simplified representation, especially if continuous characteristics are grouped into discrete categories, this is often referred to as *coarse-graining*. In the context of mixed-species groupings, individuals may not focus on which particular species is present, but rather on categorical information. For example, evaluating colour may be important in areas with predation risks, where individuals may preferentially associate with groups that match their main colour and avoid groups where they would be an unusual minority colour and could be subject to higher predation risks. All of these decisions are based on keying in to different types of information and provide different insights into which information the individuals themselves value or are able to evaluate in making decisions.

These coarse-graining methods may help animals better process the often complex information available to them to use parts of the information more easily or efficiently. Individuals may have evolved information-reduction processes like coarse-graining to collapse rich observational information into more manageable categories in order to more quickly and effectively make decisions [8]. Thinking carefully about coarse-graining is critical to taking an animal’s perspective on the kinds of information it is able to perceive, process and use [7].

Temporary species aggregations with high turnover provide a unique situation in which to test how and why individuals of one species associate with individuals of other species, and especially to investigate the categories of information these decisions may be based upon. Here, we use a suite of statistical and network methods to better understand how individuals make decisions about associating with other species. We use data from parrots that form temporary mixed-species aggregations to eat clay on exposed river banks in Peru. Individual parrots make decisions about when to visit the clay wall, where to land on the wall and when to depart. In this system, clay consumption is thought to provide necessary nutrients such as sodium, which are otherwise rare in the tropical environment [9]. Individuals interact frequently with other species at the clay, often by responding to alarm calls and aggressing against other species. Due to these interactions, individuals likely make grouping decisions to balance three factors: to access needed resources, to decrease predation risk and to avoid species that may be aggressive towards them.

We analysed data on clay lick use for 12 species of parrots. These species vary in several characteristics, most obviously in size, shape and colour. Species vary in size from very small parakeets (dusky-headed parakeet, *Aratinga weddellii*, 108 g) up to the largest macaws (red-and-green macaw, *Ara chloropterus*, 1250 g). Variability in size likely affects heterospecific interactions (e.g. aggression from larger species towards smaller species) but could also affect predation risk. Species also vary in shape from species with long tails and narrow wings (e.g. parakeets and macaws) to species with short tails and blunt wings (e.g. large parrots). These differences in body configuration affect how each species flies and their agility, which could be important for flying in mixed groups or for predator avoidance. Additionally, species vary in plumage coloration, with back colours of green, blue, or red, and facial colours of white, grey-green, yellow, orange and blue. These variations in plumage colour could be important for predation avoidance; previous research has suggested this may be important in grouping dynamics [10]. Overall, this variability in characteristics provides a range of information that species could potentially use to make decisions about which other species to associate with on the wall.

Our goal in this paper is to understand what forms of information processing, if any, are occurring between the various species that congregate on the clay lick. First, we evaluated whether these mixed-species flocks are more than mere aggregations of species that simply congregate at similar times of day and on similar spots of the clay lick to use a common resource. To answer this question, we inferred a social network of affiliations and avoidances among species that occurred significantly more than expected by chance, when controlling for species-specific preferences in clay lick usage. Second, we investigated the nature of the information that was being processed and propose several plausible categorizations systems that could explain the dynamic joining behaviour of each species. For example, is a given species’ joining behaviour better described by species-level categories, or by a system that merely discriminates between large macaws and non-large macaws? The methods we employ allow us to categorize species in different ways to test which species are potentially ‘interchangeable’ with one another and determine whether any simpler systems (with fewer parameters/categories) could explain the observed joining behaviour. Overall, a better understanding of what information individuals use to make their decisions could provide insight into the cognition underlying this decision-making process, help to better predict mixed-species grouping behaviours and provide novel insight into how and why these groupings might change.

## 2. Methods

### (a) Data sources and processing

We used observations of parrots visiting the Colpa Colorado clay lick in southeastern Peru ($13^{\circ }08^{\prime }\;S$, $69^{\circ }37^{\prime }\;W$), which is part of a long-term monitoring project. The clay lick is a 500 m long and 25–30 m tall cliff eroded out of the western bank of the upper Tambopata River. The soils of the clay lick have high levels of sodium and cation exchange capacity [11–13]. This clay lick is used by over 29 species of birds, including 18 species of parrots [14].

We used 11 years of data collected from 2002 to 2012. Observations at the clay lick take place year-round. Starting when the first birds visit the clay lick at dawn, observers at the clay lick conduct scans every 5 min. In each scan, they record the number of individuals of each species present on each area of the cliff.

To standardize location data collection over the course of this long-term project, the clay lick was divided into ‘zones’, each representing an area of the cliff where parrots use the clay. Observers were trained on the location of these zones, which were often defined by obvious landmarks. These zones are not all contiguous—some zones are separated by areas of non-usage, for example, areas with heavier vegetation cover. Zones also vary in overall size, with size somewhat dependent on the features used to more easily identify each zone. For our analyses, we focused on 11 zones that were consistently used through the long-term data collection period (zones 1A, 1B, 1C, 2A, 2B, 2C, 3A, 3B, 3B1, 3B2 and 3C). We used these zones to define co-presence during foraging. Although individuals at the extreme edges of two different adjacent zones could be closer together than individuals within the same zone, which are at opposite extreme edges within the zone, individuals observed at the same time within the same zone were generally in close proximity when foraging.

**Figure 1.**
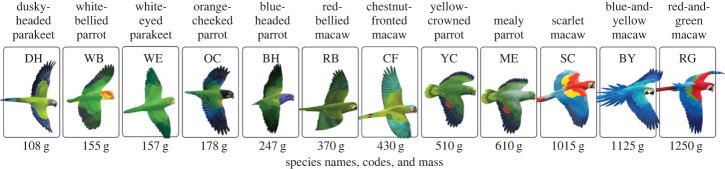
The 12 parrot species used in the analysis. The drawings depict species differences in size (shown from smallest on the left to largest on the right), coloration and body shape. For size comparison, mean species mass is shown in grams, rounded to nearest whole number [15]. Abbreviations show the two-letter codes used in the text and other figures. Species artwork by V. Darby Moore. (Online version in colour.)

We focused our analyses on the 12 species that were frequent visitors to the clay lick (figure 1): blue-headed parrot (BH, *Pionus menstruus*), blue-and-yellow macaw (BY, *Ara ararauna*), chestnut-fronted macaw (CF, *Ara severus*), dusky-headed parakeet (DH, *Aratinga weddellii*), mealy parrot (ME, *Amazona farinosa*), orange-cheeked parrot (OC, *Pyrilia barrabandi*), red-bellied macaw (RB, *Orthopsittaca manilata*), red-and-green macaw (RG, *Ara chloropterus*), scarlet macaw (SC, *Ara macao*), white-bellied parrot (WB, *Pionites leucogaster*), white-eyed parakeet (WE, *Psittacara leucophthalmus*) and yellow-crowned parrot (YC, *Amazona ochrocephala*).

We filtered the available data by species, season, time of day and zone criteria. We included only scans in which there was at least one individual of one of the 12 focal parrot species present. Because previous studies have shown that clay lick visitation varies at different times of year for different species [9], we used data from a single season for our analyses. We chose the early wet season (October to December), which is a season of high activity at the clay lick [9]. We also focused on the early morning observation periods, which is when the most species visit the clay (see electronic supplementary material, figure S1), so filtered the data to include only scans before 09:00. Finally, we excluded any scan with a non-standard zone code (that did not match the 11 zones described above) and any scans missing a date and/or time.

Prior to starting any analyses, we split the available data into two parts: one for our main analyses and one for validation. This is a common practice in data science known as cross-validation [16], which preserves our ability to test any exploratory hypotheses we develop in response to patterns observed in the first half of the dataset, as confirmatory hypotheses on the unseen half of the dataset. Practices such as these are one way to avoid ‘HARKing’ (*Hypothesizing After the Results are Known*) [17]. We partitioned the data into two halves by whether the dates of the observations occurred on odd or even days and randomly chose to use the even-numbered days as our main dataset (preserving the odd-numbered days for validation). We refer to these as the main data partition and the validation data partition, respectively. The post-processed dataset contained a total of 7357 scans in the main data partition and 7673 scans in the validation data partition. All analyses were conducted on the main data partition. Any exploratory hypotheses that were tested on the validation dataset are explicitly noted where they appear. All data processing and analyses were conducted in the R programming environment [18].

### (b) Summarizing the extent to which species mix

To get a general understanding of the amount of mixing that occurs between species on the clay lick, we computed several summary measures. First, we determined the total number of species present during each 5 min scan and report the frequency at which we observed different numbers of species together on the same zone. Then, for each focal species, we extracted the number of different species it was observed with, the group size of the focal species, the total number of individuals (focal and other) it was present with, and quantified the species diversity of the groups it was observed in using Shannon entropy in the R package *vegan* [19,20]. Last, to get an overview of each species’ level of sociality during clay lick usage, we computed the percentage of scans in which the focal species was observed in monospecific groups (i.e. the only species present on the zone).

### (c) Quantifying species-level associations using co-presence patterns

To understand which species prefer to group on the cliff with others, which species avoid each other, and what community structures, if any, result from these preferences, we inferred the overall structure of inter-species associations on the clay lick by analysing the co-presence relationships between each pair of species.

First, we quantified the total number of co-presences between each pair of species. Individuals of one species were scored as being present with individuals of another species if they were observed on the same zone during the same 5 min observation scan. For each pair of species, we counted the number of scans in which at least one individual from both species was located on the same zone at the same time. This approach used only the presence or absence of inter-species co-presences rather than the total number of individuals of each species involved. We chose this binary approach because our research questions were aimed at interspecies relationships, rather than individual relationships. Moreover, this dataset did not track identifiable individuals (i.e. we can not know if an individual observed at time *t* is the same individual observed at time *t* + 1), which precludes individual-level modelling of the social dynamics on the clay lick.

Second, we constructed a species-level association network, where each node in the network represents one species and each edge represents the type of association between two species: either affiliation or avoidance. We inferred the edge type between each pair of species by conducting a binomial generalized linear mixed effects regression analysis (glmer) using the R package *lme4* [21]. This inference procedure allows us to control for other variables, such as preferences for foraging on certain zones or at certain times of the day, which could be driving co-presences (see electronic supplementary material, figures S1 and S2) and gives us a clearer picture of the co-presence patterns that are driven purely by the presence of other species.

We conducted a glmer analysis for each species, with that species’ binary presence or absence on the clay lick entered as the dependent variable. Independent variables were the presence/absence of each of the remaining 11 species with additive effects. Random effects (for intercepts) were entered for the observation’s zone ID (e.g. 1A), time of morning (e.g. 06:00), and date (e.g. 21 October 2010). The significance of each species as a predictor for the presence/absence of the focal species was assessed by comparing a reduced model (omitting the species in question) to the full model (including the species as a predictor) using an ANOVA. Where the full model performed significantly better than the reduced model, an edge between the two species was ruled in. The type of edge was determined by the sign of the estimate, with positive estimates denoting affiliative edges (meaning the focal species was more likely to be present when the predictor species was present) and negative estimates denoting avoidant edges (meaning the focal species was less likely to be present when the predictor species was present). We corrected for multiple comparisons by controlling the false discovery rate using the Benjamini–Hochberg procedure [22] as recommended for ecological research (see [23]). Our approach with the co-presence network analysis is capable of detecting asymmetrical relationships between species (for example, species B might predict the presence of species A, but not *vice* *versa*). Each significant relationship was drawn on the network as a directed edge, *A* → *B*, where *A* was the focal species and *B* was the predictor species.

To determine whether species could be grouped by their connections within the co-presence network, we analysed the community structure on both the raw co-presence counts and the controlled co-presence associations. Analysis of the raw co-presence counts can show whether certain groups of species come into contact with one another more often than others on the clay lick, while analysis of the controlled co-presence associations can identify which groups of species actively seek to co-forage with one another. We conducted a community detection analysis on each data type using the leading eigenvector of the community matrix [24]. In the first case, each entry in the community matrix, *M*[*i*, *j*], contained the raw number of co-presence counts between the *i*th and *j*th species. In the second case, *M*[*i*, *j*] = 1 denotes an affiliative edge between species *i* and *j* after correcting for multiple comparisons and *M*[*i*, *j*] = 0 otherwise (detecting communities among the affiliative edges only).

We also quantified each species’ centrality in the affiliative co-presence network using two node-level summary measures: degree centrality and betweenness centrality. Degree centrality is measured as the total number of incoming and outgoing edges from a node [25] and betweenness centrality is calculated using the total number of shortest paths through the network that the node is located on [26]. Each of these measures captures centrality in two different senses. Degree centrality captures a local form of connectedness (how many species does one have to deal with directly), whereas betweenness captures a more global, structural form of centrality (how many species are dealt with directly and how many others end up in one’s vicinity as a result of one’s direct associations). Betweenness is commonly used to identify nodes that serve as ‘bridges’ between otherwise isolated sections of networks, or as ‘brokers’ that mediate the transfer of information among different communities within a network. All network processing, plotting, community detection and centrality calculations were done with the package *igraph* [27].

Last, after viewing the resulting co-presence network that we constructed using the main data partition, we noticed that the body size differences between species seemed to be predictive of their edge type, with avoidant relationships being more likely to occur between species with larger differences in body size. Because this was an unanticipated relationship in our original analyses, we treated it as a new hypothesis and tested it in the validation data partition. For each pair of species involved in a significant edge, we computed the difference in the average body mass between those species (figure 1). We tested this directional relationship (whether large differences in body size were more likely among avoidant edges) by conducting a one-tailed Mann–Whitney *U* test on body mass differences between the two groups of edge type (affiliative and avoidant). We used this approach to identify a significant relationship in the main data partition, then tested whether this association replicated in the validation data partition.

### (d) Quantifying dynamic joining patterns

While co-presence patterns can inform us about co-foraging preferences among species, these patterns are the aggregate product of several species’ dynamic decision-making preferences. To gain a clearer picture of each species’ individual decision-making process, we leveraged the fine-grained temporal aspect of this data (with scans made every 5 min) to extract all joining events among species. This dataset offers a unique opportunity to investigate the fine-grained temporal dynamics because about 90% of the observation scans occurred in runs of regular 5 min intervals. From these runs, we extracted each species’ joining behaviour and inferred the significant joining relationships among species.

**Figure 2.**
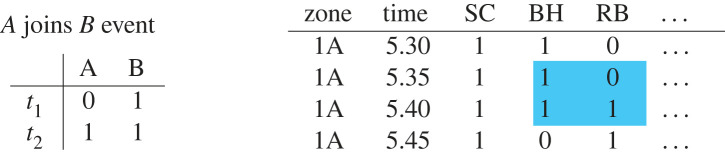
Example joining events extracted from dataset of binary presence or absence of species. In the left example, species A is absent at *t*_1_, but *joined* species B at *t*_2_. The right example shows a joining event from the main data partition, highlighted with the blue box. RB was absent at 5:35 in zone 1A while other species were present. RB *joined* BH (and SC) in zone 1A at 5:40. (Online version in colour.)

We considered joining events between pairs of species, *A* and *B*, across two 5 min adjacent scans, *t*_1_ and *t*_2_. We defined the event *A* joins *B* as occurring when *B* was present at both *t*_1_ and *t*_2_ and *A* was absent at *t*_1_ but present at *t*_2_ (figure 2, left). Figure 2 (right) shows one example of a joining event as it appears in the dataset, where RB joined BH at 5:40.

We constructed a network of joining behaviour between each pair of species using the same procedure described in the previous section for the co-presence network, but for different dependent and independent variables. The dependent variable was whether or not the focal species (in role *A*) had just joined the zone (e.g. the dependent variable for RB in figure 2 would read 0,0,1,0). The independent variable for each of the 11 predictor species showed whether or not the species (in role *B*) was present at *t*_1_ and *t*_2_ (e.g. the independent variable for BH in figure 2 would read 0,1,1,0). We inferred the presence of significant edges using the binomial glmer method, as outlined in the previous section, and corrected for 132 multiple comparisons with the Benjamini–Hochberg procedure. Edges are directed and colour-coded to indicate affiliative or avoidant joining behaviour: a blue *A* → *B* means that *A* was significantly more likely to join the zone if *B* was present and a red *A* → *B* means *A* was significantly less likely to join if *B* was present. The inferred network of joining behaviour can determine whether an observed co-presence edge between species was the product of reciprocated or unreciprocated joining preferences between those species.

To determine whether species could be grouped by their connections within the joining network, we also conducted a community detection analysis using the same methods as described in the previous section. The community matrix contains a 1 where an affiliative join edge exists between two species, −1 for an avoidant join edge and 0 for any edge that did not qualify as significant after correcting for multiple comparisons.

### (e) Identifying potential categorization schemas

Although species-level information is a convenient way for us to summarize co-presence and joining patterns, that information may not be what the parrots are using when deciding which co-foraging partners to join. They may instead be using less detailed information and join others on the basis of more general categories like colour or body size. This process is known as ‘coarse-graining’ [6–8], where more detailed information (like species categories or the absolute size of individuals) are lumped together into larger, less specific categories (like general colour categories or size classes). Because information processing is costly, evolution and learning alike tend to favour parsimonious rules and heuristics over redundancy in encoding procedures [8,28].

We constructed several plausible coarse-grained categorization systems and tested whether they outperform the species-level system when fit to the data on joining behaviour. We refer to each of these as ‘categorization schemas’ (which describe how sets of species were grouped) and use ‘categories’ to refer to the identifying feature of a particular set. For example, a *categorization schema* may be based on colour, with red, blue and green representing the *categories* within that schema. All red-coloured species would then be assigned to the ‘red’ category in this categorization schema. We focus on categorizations because we used a coarse-graining approach, which requires binning of detailed information into less-detailed larger categories, and because we tested whether any of these methods could outperform a species-level categorization system to explain association or joining patterns in the parrots.

We tested nine categorization schemas within three feature domains: body shape, colour and size. Table 1 shows how we grouped species under each of the nine categorization schemas. (1) Grouping by species. (2) Grouping by clade and general shape, which could affect flight manoeuverability and a species’ ability to evade aerial predators [10]. (3) Grouping by whether parrots are large macaws or not, because the large macaws are much larger than all the other parrots and could potentially be highly aggressive to smaller species. (4) Grouping by majority back body colour, which is the most obvious colour when birds are perched on the wall and could be used both by other parrots to make joining decisions as well as by predators. (5) Grouping by rear head colour, which could make species distinctive in flocks and could be used by both other parrots and predators. (6) Grouping by distinctive face colour, which may be used as a social signal when parrots are nearby each other on the wall. (7) Grouping by size, which serves as a further coarse-graining of clade. (8) Grouping by whether or not the species is larger or smaller than the focal species, determined by the average body mass of the species. (9) Grouping by whether or not the species is similarly sized to the focal species, where the species immediately adjacent in size to the focal species were labelled as similar and all others were labelled as different. The three size systems account for the possibility of size-based aggression [10] and the final two size systems were defined relative to the focal species.

**Table 1.** Description of the nine categorization schemas. Columns show how species were grouped into categories under each categorization schema. Numbers in parentheses in column headings show the numbers of categories for each categorization schema. The last two schemas (identified with *) are relative to the size of the focal species, and are demonstrated here by using BH as the focal species.

species	clade	large macaw	back colour	head colour	face colour	size	larger*	similar*
(12)	(5)	(2)	(3)	(5)	(5)	(3)	(2)	(2)
DH	parakeet	no	green	green	green-grey	small	smaller	different
WB	small parrot	no	green	orange	orange	small	smaller	different
WE	parakeet	no	green	green	green-grey	small	smaller	different
OC	small parrot	no	green	black	yellow	small	smaller	similar
BH	small parrot	no	green	blue	blue	small	(focal)	(focal)
RB	small macaw	no	green	green	white	medium	larger	similar
CF	small macaw	no	green	green	white	medium	larger	different
YC	large parrot	no	green	green	yellow	medium	larger	different
ME	large parrot	no	green	green	green-grey	medium	larger	different
SC	large macaw	yes	red	red	white	large	larger	different
BY	large macaw	yes	blue	blue	white	large	larger	different
RG	large macaw	yes	red	red	white	large	larger	different

Our main goal with the category analysis was to investigate whether simpler and more coarse-grained categorization schemas outperformed the more complex and information-rich species-level categorization schema. We based our categorization schemas on a range of biologically relevant factors that are plausible alternatives a species-level encoding system. Shape- and size-based systems are relevant to inter-species competition and aggression on the clay lick, whereas colour-based systems are relevant to predation risk (e.g. a green parrot may only join other green parrots to avoid being obvious to predators). For the colour-based schemas, it is important to note that many birds use UV signals that are outside of the perception range of human visual systems [29,30]. The birds could be using a UV-based colour schema that was not captured in our analysis. Unfortunately, we do not have a good understanding of the specific UV signals of all 12 species in our dataset and were unable to use UV signals as the basis for a categorization schema. The nine categorization schemas used here should not be interpreted as an exhaustive exploration of all the possible ways in which the birds could be making their joining decisions. The birds may be using another categorization schema not investigated here, so this analysis cannot be used to definitively determine which particular schema each species uses. However, our main goal was to compete the species-level schema against other simpler and biologically plausible schemas, a question that our approach can address.

For each species, we inferred which of the nine models best described that species’ joining decisions. We constructed a set of nine nested binomial glmer models, where each model encoded one categorization schema, and competed these models against one another in an Akaike information criterion (AIC) model comparison framework. Each model was fit to the joining data and defined as described in the previous section. The independent variables, however, were modified to encode each categorization schema. The encoding procedure worked by creating a new variable for each category. For example, for the clade categorization schema, any species that was classified into the category ‘small parrot’ was assigned a 1 in the main data partition for each observation if any of the 11 predictor species of the type ‘small parrot’ were present (at *t*_1_ and *t*_2_), and assigned a 0 otherwise.

We compared these nine models using an ANOVA and identified the model with the lowest AIC as the best-fit model [31]. Where the second best model had a Δ*AIC* > 2, we report both models. In general, models have lower AIC if (1) they describe the data better and (2) they have fewer parameters [32], so this procedure allowed us to identify cases where lower-complexity categorization schemas outperformed the higher-complexity species schema. We computed the Akaike weight of each model, which gives the probability that each model is the best-fit model, given the set of candidate models and the data [33]. We reported the Akaike weight of the best-fit model and the species model to give a sense of our confidence in ruling in the simpler model over the species model. We summarized the results of each best-fit model to understand how the species relate to one another (in terms of avoidance or attraction) within the inferred categorization schemas.

For each species, once we had identified the best-fit categorization schema, we identified which categories within the schema the species significantly preferred, significantly avoided, or joined at statistically non-significant levels. Using these categories, we mapped these positive, negative and neutral relationships back onto species identities according to which category they fell into (table 1).

Finally, we quantified the usage complexity of the best-fit categorization schema for each species. We defined ‘usage complexity’ as the number of categories that each species significantly preferred or avoided in making joining decisions within the best-fit categorization schema. For example, a species might have a best-fit with the *back colour* schema, which contains three categories that could potentially be preferred or avoided in making joining decisions (green, red and blue). If the species significantly preferred to join green- and red-backed birds, but significantly avoided blue-backed birds, the usage complexity would be 3. However, if the species only significantly preferred to join green-backed birds and did not significantly prefer or avoid red- and blue-backed birds, the usage complexity of the *back colour* schema for this species would be 1, because it only significantly responded to one category out of the three potential categories in the schema.

## 3. Results

### (a) Summarizing the extent to which species mix

**Figure 3.**
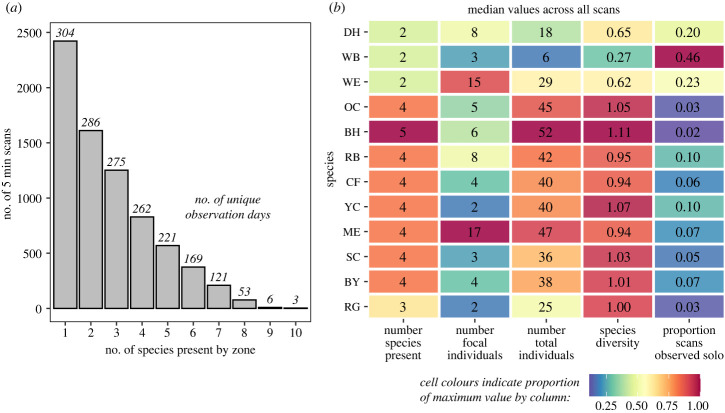
Summaries of the extent of mixed-species aggregations. Panel (*a*) shows the numbers of scans with groups comprised of different numbers of species within zones (with the number of unique sampling days shown above each bar). Panel (*b*) summarizes how often each species participated in mixed-species groupings within zones. For each focal species, columns show: (1) the median number of species present in a zone during a single scan when each focal species was present, (2) the median number of focal individuals present during each scan at each zone, (3) the median total number of individuals present during each scan at each zone (across both the focal species and all other species), (4) the median species diversity in groupings during each scan at each zone that each focal species was present in, and (5) the proportion of scans where the focal was the only species present on the zone. Cell colours indicate each value’s proportion compared to the maximum value per measure for each column, with red shades highlighting the maximum values for each column. (Online version in colour.)

We documented a high degree of interspecies mixing on the clay lick. In the main data partition, 67% of scans (4935 observations) recorded individuals from more than one species present at the same zone at the same time, with a median of 2 and a maximum of 10 species present together (figure 3*a*). Species differed in the extent to which they mixed with others (figure 3*b*). For example, blue-headed parrots displayed the highest levels of interspecies mixing: they were observed with the highest numbers of species, the largest total numbers of individuals, and in the highest species diversity of groupings, although generally with small numbers of conspecifics. By contrast, white-bellied parrots displayed the lowest levels of interspecies mixing: they were observed with the lowest numbers of species, were present in groupings with the lowest species diversity, and were most likely to use the clay lick in monospecific groups.

### (b) Co-presence patterns

**Figure 4.**
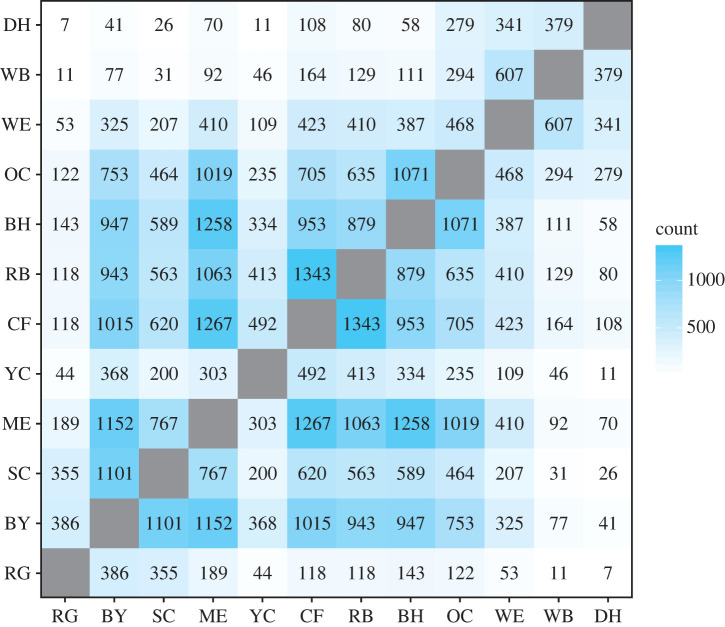
Raw co-presence counts between each pair of species. Each cell shows the number of observations in which the two species were present on the same zone at the same time. The two species that occurred together the most were RB and CF (1343 scans) and the least were DH and RG (seven scans). Axes show species ordered by body size, from DH (smallest) to RG (largest). (Online version in colour.)

Using the raw counts of co-presences in the main data partition, we found variability in how often each pair of species was observed together (figure 4). Using a community detection algorithm on these raw counts, we found evidence for two communities within the raw co-presence network: one community among the four smallest species (DH, WB, WE and OC) and another among the eight largest species (BH through RG). While this result suggested that interspecies usage of the clay lick was structured, this structure could have been the product of common preferences for foraging on particular zones at particular times of the day, rather than direct preferences for associating with certain species. To understand these potentially confounding variables, we extracted species-specific trends in clay lick usage by zone and time of morning and describe these in the electronic supplementary material, §SI1. We found little variation in clay lick usage across species by time of morning, with the exception of the three large macaws, which returned to the clay lick in the late morning (electronic supplementary material, figure S1). For zone preferences, however, we found two clear usage regimes that may explain the community structure detected above: the three smallest species (DH, WB, WE) tended to forage on zone 1A and the remaining larger birds (including OC) tended to forage in zone 2C (electronic supplementary material, figure S2).

**Figure 5.**
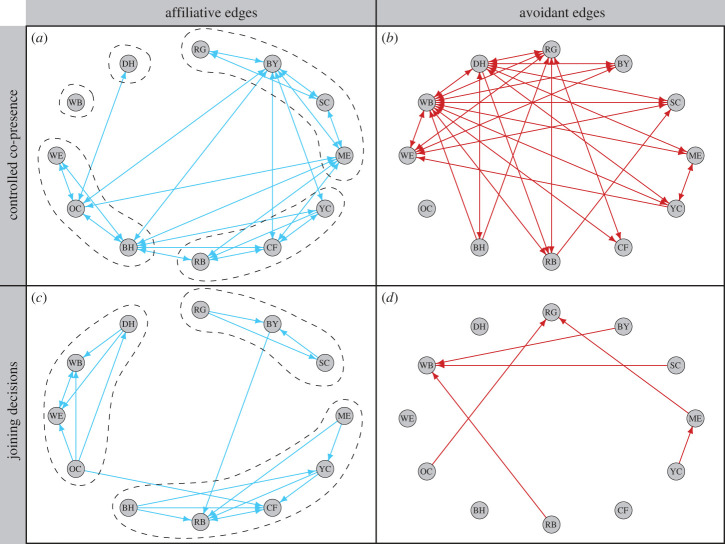
Networks of co-presence and joining among parrot species, showing affiliative (blue) and avoidant (red) edges among species pairs. The top two panels show the network inferred from the controlled co-presence data: (*a*) affiliative edges indicate species that occurred together on the same zone of the clay lick significantly more than expected by chance, while (*b*) avoidant edges indicate species that occurred together significantly less than expected by chance. The bottom two panels show the network inferred from the joining data: (*c*) affiliative edges, where *A* → *B* shows species *A* was significantly more likely to join a zone if *B* was already present and (*d*) avoidant edges, where *A* → *B* shows species *A* was significantly less likely to join a zone if *B* was already present. Dashed lines in panels (*a*) and (*c*) show shared community membership, identified from community detection on the two types of affiliative networks. Nodes in all four networks were arranged by body side, running clockwise from RG (largest) to DH (smallest). (Online version in colour.)

To better understand species co-presence patterns, we then controlled for the effects of zone and time of morning on associations between species pairs. We used these controlled associations to construct a network of significant affiliations and avoidances among species on the clay lick (figure 5*a*,*b*). We tested all 132 potential species associations to identify significantly attractive and avoidant associations.

We found that the majority of co-presence edges were reciprocated when present, showing that if species A preferred or avoided B, species B also usually had the same relationship with A. Only 1 positive edge was unreciprocated and five negative edges were unreciprocated.

Using community detection on the now-controlled co-presences, we identified five communities in the network of the affiliative edges: a group involving the large macaws (RG, BY, SC, ME), a group involving the small macaws (YC, CF, RB), a group of the small parrots (BH, OC, WE), an isolate group for WB and an isolate group for DH (figure 5*a*).

We also found that some species were more central than others in the affiliative co-presence network. Across the 12 species, BH, BY, ME and OC had the highest betweenness centrality, indicating that they connected different communities within this network and could have functioned as potential ‘brokers’ of co-foraging affiliations. These same four species, with the addition of CF, also had high degree centrality, meaning they were directly connected to the highest number of species. (Centrality scores for each species can be found on the *x*-axes of figure 8).

**Figure 6.**
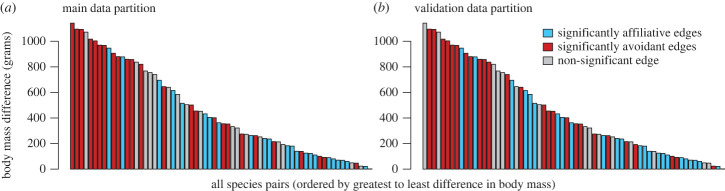
Body mass differences explained part of the variation in controlled co-presence association types. Negative associations were more likely to occur between species with a larger difference in body mass and positive associations were more likely to occur between species with similar body mass. Bars show the difference in body mass between all possible pairs of species, ordered from largest to smallest: blue bars show edges that were significantly affiliative in the controlled co-presence network (figure 5*a*) and red bars show edges that were significantly avoidant (figure 5*b*). We found a significant association between body mass differences and the type of association (affiliative/avoidant) in the main data partition (*a*), then tested for and found the same pattern in our validation data partition (*b*). The mean body mass of each species is shown in figure 1. (Online version in colour.)

In our analysis of the main data partition, we found that the type of co-presence association between two species (affiliative or avoidant) could be partially explained by size differences between pairs of species. Species with larger size differences tended to have negative associations, while species that were more similar in size tended to have positive associations (figure 6). In the main data partition, the mean difference in body size was 301 g for positive edges and 608 g for negative edges. A Mann–Whitney *U* test found that these two groups differed significantly (*U* = 1538, *n*_1_ = 45, *n*_2_ = 47, *p* < 0.001, one-tailed). We then tested for this same association in the validation data partition and detected the same pattern (mean difference in body size was 296 g for positive edges and 599 g for negative edges; Mann–Whitney *U* test showed these two groups differed significantly: *U* = 1671, *n*_1_ = 47, *n*_2_ = 48, *p* < 0.001, one-tailed). These results provided evidence that species' body size differences affect their co-foraging relationships.

### (c) Dynamic joining decisions

We examined attraction and avoidance from the joining decisions of species (figure 5*c*,*d*). When we filtered the data to include only sequential runs of 5 min adjacent scans, we found that the main data partition included 1289 separate runs of 5 min adjacent scans, representing a total of 6586 five min scans. We used these sequential scans to assess joining patterns.

Networks of affiliative and avoidant species associations were much less dense in the joining networks compared to the controlled co-presence networks, with many fewer significant edges between species. These edges were also far less reciprocal than in the co-presence networks. Overall, the joining network contained a striking amount of directionality, with only two pairs of species exhibiting reciprocal joining preferences (RB–CF and WE–WB) (figure 5*c*).

The differences between the joining network and the controlled co-presence network allowed us greater insight into the types of associations between species. For example, we found that the relationship between the three large macaws was more structured when we considered joining decisions rather than simple co-presence. We found that RG joined BY and SC, SC only joined BY, and BY actively joined neither of the large macaw but instead joined the small macaw RB. We also found a directed, hierarchical structure to the joining behaviour in the small parrot cluster: OC joins WE, WB and DH, DH joined WE and WB, whereas WE and WB only joined one another. We found that the strong, multi-species avoidance of WB was driven by larger species (BY, SC, RB) not joining WB, rather than WB not joining them. The joining network also allowed us greater insight into the anomalous avoidant co-presence edge between two adjacently sized species ME and YC (figure 5*b*): ME joined YC, as would be expected from similarly sized species (figure 5*c*), however YC avoided joining ME (figure 5*d*). Last, we found that the largest species, RG, was avoided by smaller species, and not the other way around.

When we used community detection on the joining network, we found that the joining network was divided into three main communities (figure 5*c*): one involving the large macaws (RG, BY, SC), one involving the small macaws and large parrots (ME, YC, CF, RB, BH), and one involving the small parrots and parakeets (OC, WE, WB, DH).

### (d) Inferring categorization schemas from joining patterns

**Figure 7.**
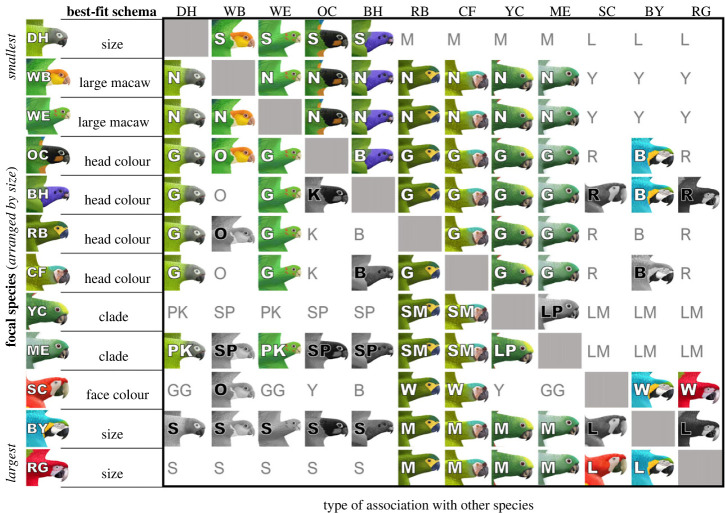
Best-fit categorization schema models by species for dynamic joining decisions. Each focal species is labelled in the first column, followed by the best-fit model inferred from that species’ joining patterns. All best-fit categorization schemas, with the exception of those for WB and WE, had Δ*AIC*s > 2, making them strongly differentiated from the next-best model (see electronic supplementary material, table S1 for AIC results). Across the table, species that the focal significantly prefers are shown in colour with a white category label, while species they significantly avoid are shown in greyscale with a black category label. Cells containing only text indicate species with which the focal did not have a significantly positive or negative association. All cells are labelled with a text code that indicates the category that each species falls into under the best-fit categorization model (*size*: S, small; M, medium; L, large; *large macaw*: N = is not a large macaw, Y = is a large macaw; *head colour*: G, green; O, orange; K, black; B, blue; R, red; *clade*: PK, parakeet; SP, small parrot; LP, large parrot; SM, small macaw; LM, large macaw; *face colour*: GG, green−*grey*; O, orange; Y, yellow; W, white; B, *blue*). See table 1 for species assignments to categories. Species artwork by V. Darby Moore. (Online version in colour.)

We tested whether a simpler categorization schema, other than species, could better explain each species’ joining patterns. For all 12 species tested, a simpler categorization schema beat out the species-based schema by a large margin (figure 7). Electronic supplementary material, table S1 shows the Akaike weights for the best-fit model (*wAIC*_best_) compared to the species model (*wAIC*_species_). Across all cases, the species model performed with a *wAIC* of 0.003 or less, meaning that the probability that the species model was the best-fit model, given the nine categorization schemas tested and the data, was never higher than 0.3%. We tested whether this result—that species used simpler coarse-grained rules to make joining decisions—replicated in the validation data partition. Our results support our main conclusion about information processing in this system: 11 of our 12 species had a best-fit categorization schema that was simpler than the full species schema. The only exception to this pattern was OC, for which the species schema was the best-fit model. OC made significant use of seven of the species schema’s categories (it joined BH, CF, DH, RB, WB, WE and YC). In the main data partition, the best-fit schema for OC was headcolour.

After ruling out the species-based schema, we looked at each species' best-fit schema and saw that these varied considerably by species (figure 7). The absolute *size* schema was the best-fit model for DH, BY and RG. *Head colour* was best for OC, BH, RB and CF and *face colour* was best for SC. *Clade* was best for ME and YC and the *large macaw* model was best for WB and WE (with the *back-colour* model coming in as a close second). Interestingly, neither of the relative size models was the best-fit model for any species, despite body size being predictive of edge type at the network level.

We also evaluated whether each species showed consistent evidence of use of a particular categorization schema by comparing the best-fit schema in the main data partition to that found in the validation data partition. We found that the best-fit categorization schema was the same across the two data partitions in the majority of species. Of our 12 species, eight had the same best-fit categorization schema in the validation data partition as we found in the main data partition. While our investigation of potential categorization schemas was not exhaustive, the fact that the best-fit categorization schema of these eight species was consistent provides additional support for the biological relevancy of these schemas. Four species had a best-fit schema that differed from our main results: in the validation data partition, WB and CF returned *size*, RB returned *larger*, and OC was best-fit by *species*.

In the main data partition, we found good evidence for a preference for similarity: 10 to 11 of the 12 species exhibited a significant preference for joining others that were categorized in the same category as themselves within the best-fit schema (electronic supplementary material, figure S4). One species, the orange-cheeked parrot, preferred head colour but had no potential same-coloured birds it could join as it was the only species with a black head. Of the total species, only the yellow-crowned parrot showed significant avoidance of a species in its own category: the clade schema was the best-fit model for this species and it belonged to the large parrot category, however it avoided mealy parrots, which were the only other large parrot. We also found some preferences for joining individuals belonging to different categories: 5 of the 12 species preferred to join individuals in other categories from their own. Finally, we found that half the species significantly avoided at least one category; for 5 of the 12 species, this avoided category was something other than the one the focal belonged to. Together, these results provide mixed evidence for phenotypic assortment or trait matching. Under strict assortment, we should see evidence of both a preference for similarity and avoidance of differences. Here, we see consistent evidence for similarity preferences but weaker and mixed evidence for avoidance.

**Figure 8.**
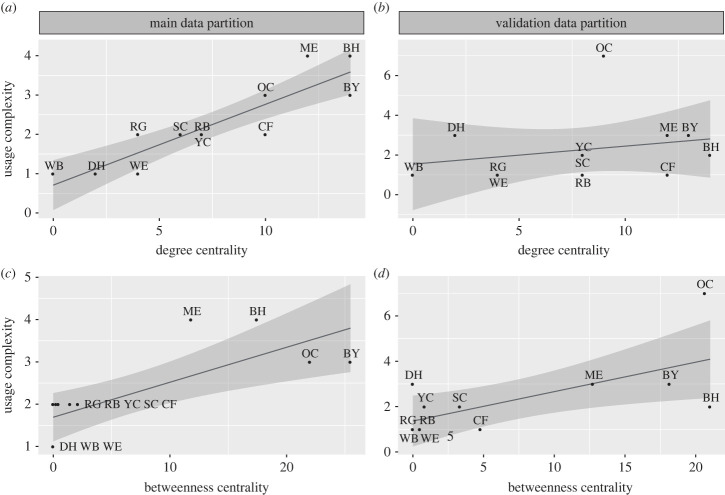
The relationship between usage complexity of the best-fit schemas for each species and centrality in the co-presence network. The *y*-axis is ‘usage complexity’, the number of statistically significant categories within the best-fit categorization schema that each species used when joining other birds on the clay lick. The *x*-axis shows each species’ degree centrality (*a*,*b*) and betweenness centrality (*c*,*d*) in the inferred co-presence network. We found degree and betweenness were both significantly predictive of usage complexity in the main data partition (*a*,*c*), but only betweenness remained significant when tested in our validation data partition (*b*,*d*).

When we examined the usage complexity of the best-fit categorization schemas per species, we noticed that species that were making significant use of a higher number of categories (figure 7) were also more central in the affiliative co-presence network (figure 5). In the main data partition, degree and usage complexity had a significant positive relationship (estimate = 0.206, s.e. = 0.033, *t*-value = 6.272, *p* < 0.001, figure 8*a*). The addition of one degree to a species centrality score led, on average, to the use of 0.206 more categories. Betweenness and usage complexity also had a significant positive relationship (estimate = 0.083, s.e. = 0.022, *t*-value = 3.708, *p* = 0.004, figure 8*c*). Each additional betweenness point led to the use of 0.083 more categories. We tested whether these results replicated in the validation data partition and found weaker and mixed support for a relationship between a species’ position in the social network and the usage complexity of the best-fit categorization schema. In the validation partition, the effect of degree was not significant (estimate = 0.091, s.e. = 0.116, *t*-value = 0.783, *p* = 0.452, figure 8*b*). However, betweenness was significantly positively related to usage complexity (estimate = 0.130, s.e. = 0.047, *t*-value = 2.782, *p* = 0.019, figure 8*d*). In the validation partition, an increase in each betweenness point led to the use of 0.13 more categories.

## 4. Discussion

In this paper, we described the mixing of 12 species of parrots as they formed groups to ingest clay from a riverbank in Peru. We report on two main results. First, we found evidence that species co-presences and joining decisions were non-randomly structured, showing that association patterns in this system can be treated as mixed-species groupings rather than simple aggregations. Second, we evaluated information processing patterns contained within joining decisions. We found consistent evidence that the parrots likely used a simpler categorization schema, rather than differentiating each other as species, when choosing which groups to join. Together, these results provide new insight into the grouping decisions of parrots in this system, but also provide a novel approach that could be used to detect different kinds of information processing in other mixed-species groupings.

### (a) Evidence for mixed-species associations and groups

Our first main result was support for treating this system as a mixed-species group rather than a simple aggregation of species. We documented high levels of species mixing while parrots foraged for clay on the cliff. These results are consistent with previous results at the same site, which estimated that 92% of lick use occurred with a mix of species [10].

Gatherings of individuals around a specific location or resource are generally treated as mixed-species *aggregations* rather than mixed-species *groups* [1,34]. The behaviour we observed of the parrots visiting a clay lick cliff could have represented a simple aggregation of species around a resource they are all attracted to. In this area of Peru, the parrots do not tend to aggregate with other species away from the cliff except in some cases at fruiting trees [10,35], indicating that their general behaviour is different when they visit the clay. However, simple attraction to the clay as a resource cannot explain this aggregating behaviour because the cliff is quite large and the soil appears usable across the majority of the lick. The dense aggregations of individuals we observed suggest that there is pressure to congregate rather than spread out to better avoid other species [10].

In addition, we found significant structuring in species-level preferences for co-foraging partners. These cannot be attributed to simple species-level differences in preferences for foraging at certain times of the day or on certain areas of the cliff. Our statistical approach allowed us to control for these potential preferences that might have affected co-foraging associations. Using this controlled approach, we still found significant affiliative and avoidant relationships between species. The results of this analysis show that the presence of certain species indeed affected whether other species will join and remain on the clay lick. This means that a certain amount of information about the identity of different species was being processed by these parrots and that these mixed-species flocks were not simple aggregations. At minimum, these results show that the parrots represent mixed-species associations rather than simpler aggregations around a resource.

To evaluate whether the parrots qualified as a mixed-species *group*, we examined the interactions among individuals of different species [1,36]. In our dataset, we used active joining of groupings as a measure of decision-making. We do not have data on other types of interactions in this dataset, but the parrots frequently responded to each others’ alarm calls, although previous work at the site showed that over 90% of these group flushes have no apparent cause, indicating the possibility for a high rate of false alarms [10]. Parrots were also frequently aggressive towards each other, with larger species usually displacing smaller species [10]. Taken together, these results show several lines of evidence that the parrot species likely qualify as mixed-species groups. Groupings of species on the cliff were not mere aggregations that were the outcomes of similar spatial, temporal or resource preferences. Rather, the mixed-species groups of parrots represented more complex interactions among species driven by active attraction or repulsion to particular species. Future research on directed aggression between species or alarm calling patterns and flush responses between species could provide additional insight into the nature of species relationships.

Finally, our results also provide insight into the costs and benefits of participating in particular groups. Groups composed of multiple species were commonly observed and species were rarely observed eating clay in single-species groups during the early morning hours on the cliff, even though the cliff offers ample room to do so. This suggests that joining groups could provide benefits to the parrots. However, we also found evidence for some potential costs. The three smallest parrots were observed in the most monospecific groupings. Interspecific aggression in this system tends to follow body size [10], so these smallest species may be at the highest risk of aggression on the cliff. Overall, these results show that the benefits of joining mixed groups likely outweighs the potential costs, but interspecific relationships were still somewhat structured to mitigate these costs. More study is needed to determine whether the associations we found to be negative, avoidant, or aversive are actually tied to more aggression between those species.

Our results also highlight that the ways that species associations are quantified and categorized can strongly affect our perception of the mixed-species group dynamics. In our results, we found striking differences between species associations when we compared static co-presence networks to dynamic joining networks. We argue that quantifying joining behaviour provided a clearer window into each species’ decision-making. Whereas the co-presence network was an aggregate product of several species’ decision-making, the joining behaviour network showed the direct choices made by each species.

Across species, red-bellied macaws and chestnut-fronted macaws were typically the first species to land on the clay lick in the morning, and therefore might have been prime partners to join for that reason (see electronic supplementary material, figure S3). Interestingly, although red-bellied macaws commonly initiated the coalescence of a group on the cliff, they were not one of the central species in the association network. Mealy parrots also exhibited the longest average run lengths on the clay lick (being present for an average of 4.6 consecutive scans) and this may have also made them a common species to join.

The joining decision network also provided a clearer picture of sub-community structure in these species. Previous analyses of parrot mixed-species groupings at the same site were based on co-presence and closely match the five communities detected in our co-presence network [10]. In the joining network, however, we found a coarser structure that was consistent with biological clade divisions: one group among the large macaws (RG, BY, SC), a second group among the small macaws and large parrots (ME, YC, CF, RB, BH) and a third group among the small parrots and parakeets (OC, WE, WB, DH). It is also important to note that clay lick use in the region varies greatly by season, with relative abundance and relative time of use varying among and within species across seasons [9]. Further insight into species associations could be gained by determining whether the parrots consistently prefer and avoid the same species across seasons because seasonal variation in relative abundance may make preferred or avoided species rarer or more common at the cliff, which may alter joining decisions.

### (b) Evidence for information processing within mixed-species groups

Our second main result was that we discovered several lines of evidence showing information processing in these mixed-species groups. Simply controlling for zone and time preferences and still finding structure in co-presence networks demonstrates that the parrots were processing information about other individuals rather than just acting on these preferences. The inferred co-presence network contained different information from the raw co-presence counts: whereas raw co-presence counts showed how often different species come into contact with one another while foraging on the clay lick, the inferred network showed active co-foraging relationships among species and revealed more complexity in these social relationships. When we considered the temporal dynamics on the clay lick, we found additional structure in how species’ joining decisions were based on the presence of other species at the cliff. Finally, our categorization method, where we assessed multiple potential categorization schemas the parrots could be using to make joining decisions, provided additional evidence for higher-level information processing in this system.

By competing multiple potential categorization schemas against each other, we were able to rule out a species-level categorization schema for all of the species we tested. Instead, we found evidence suggesting the species could have been using more coarse-grained categorization schemas to make joining decisions, like focusing on distinctive features such as size or colour rather than species identity. Although the set of categories we tested were by no means exhaustive, we found that simpler categorization schemas were much more strongly supported than a species-based schema. This result suggests that the information these parrots are processing about one another as they decide when and where to join the clay lick was not necessarily discriminatory at the level of biological species.

Across all our analysis methods, we found that no one species was uniformly attractive or repulsive to all other species involved in the groups. This shows that for these parrots, the species should not be categorized as serving a single functional role within these mixed-species groups. When categorization approaches have been used in mixed-species groups, the goal has been to find the general category that each species falls into to define its role in the group (such as catalysts, or initiating/core/central/nuclear/important species; reviewed in [2]). Our approach provided us with the flexibility in our analyses to find a consistent role if one existed, but also allowed each species to differ in how they associated with or categorized other species.

Previous work has shown that species may use phenotypic similarity or trait matching to choose which individuals to associate with in mixed-species groups, for example preferring to associate with others that match their own general size [37–39] or plumage colour [39]. More generally outside of mixed-species group contexts, this effect is known as self-referent phenotype matching and is used by some species in kinship recognition and mate choice (reviewed in [40]). Within our mixed-species groups of parrots, we found mixed evidence for phenotype matching. While our results showed that the parrots nearly always significantly preferred to join groups containing other species belonging to their own category, we also found that other categories that did not match the focal’s own were also preferred by almost half of the species. We show that just under half of the species avoided others assigned to a different category. Under strict phenotype matching, species should prefer others similar to themselves and avoid those that differ. Our results show a strong and consistent preference for similarity, but also some preference for non-matching individuals and no consistent strong aversion to dissimilar individuals.

Finally, we used our categorization schema results to assess the potential complexity of information processing involved in the joining decisions and found that the parrots differed in the complexity of the categories they could have used to make these decisions. Our categorization schema model fitting approach allowed us to identify the simplest kinds of information that each species could have used to make observed association decisions. We found little evidence supporting a *species* schema, and interestingly, found evidence that several different categorization schemas were in usage within this mixed-species group. In five species, the best model was a categorization schema involving *head* or *face colour*, each of which included five categories. In other species, shape- and size-based category systems were best. The two largest species (RG and BY) and the smallest species (DH) were the only species where general *body size* was the best-supported categorization schema, which involved three categories.

A better understanding of how each focal species may group other species into categories would provide insight into the sets of species that may be considered functionally interchangeable associates. Hypotheses about why these species may be functionally interchangeable could then focus on social, ecological and cognitive factors. For example, categorization schema results could be paired with classic cognitive testing to determine whether a species treats others as interchangeable because perceptually it cannot tell the difference between individuals of different species. Similarly, testing for patterns during different times of year, when the ecological factors such as food availability may be different, could provide insight into whether categorization systems are robust to changes or flexible and responsive. Focusing future work on behavioural interactions between species or sets of species could provide insights into the social costs or benefits of associating with other species.

In addition to finding best-fit categorization schemas that differed in complexity, with some having more categories than others, the joining decisions also allowed us to detect how each species actively used the different categories within their schema. We found some evidence of a positive relationship between the number of categories each species seemed to be using and the centrality of each species in the affiliative co-presence network: in both the main and validation data partitions, this relationship was significantly positive for betweenness centrality, but was only significantly positive for degree centrality in the main data partition, not the validation data partition. This form of relationship between network position and complexity of social decision-making is predicted by theories such as the social brain hypotheses [41], which states that complex social environments place high information-processing demands on individuals, which encourages the evolution or development of increased complexity in cognitive abilities. This could create pressure for parrots with more inter-species associations to encode a higher number of distinctions among species when navigating their social environment and deciding when and where to join the clay lick.

### (c) Conclusion

Association patterns in mixed-species flocks can be complex and multifaceted. The types of attraction and avoidance patterns each species follows can also differ across species. Our methods allowed us to parse inter-species co-foraging relationships in several ways, using techniques from statistics, social network analysis and cognitive science to better understand the types of connections between species, from the perspective of each species involved. Overall, our results provide evidence that simpler heuristic rules or categorization schemas (other than species identity) likely drove co-foraging decisions in these mixed-species parrot groups. Our results also show that careful consideration of not just association types, but also the ways in which species may categorize each other and how available information is coarse-grained, is likely to provide additional insight into mixed-species groupings. A more nuanced approach to identifying how and why species interact with other species could help us better understand the costs and benefits of these associations and how changes in conditions may alter the cost–benefit ratios.

## Data Availability

All data and code are available on GitHub at https://github.com/vanferdi/birds-on-the-wall. Data are available from the Dryad Digital Repository: https://doi.org/10.5061/dryad.b5mkkwhhv [42].
